# Conditional deletion of *Cc2d1a* increases irritability-like behavior by decreasing oxytocin expression via the prelimbic-hypothalamic pathway

**DOI:** 10.1186/s12929-026-01263-w

**Published:** 2026-06-01

**Authors:** Kuan-Hsiang Cheng, Yu-Chieh Hung, Pin Ling, Kuei-Sen Hsu

**Affiliations:** 1 Institute of Basic Medical Sciences, College of Medicine, National Cheng Kung University, Tainan, 70101 Taiwan; 2 Department of Pharmacology, College of Medicine, National Cheng Kung University, No. 1, University Rd., Tainan, 70101 Taiwan; 3 Department of Microbiology & Immunology, College of Medicine, National Cheng Kung University, Tainan, 70101 Taiwan; 4 Neuroscience Center, College of Medicine, National Cheng Kung University, Tainan, Taiwan

**Keywords:** *Cc2d1a*, Irritability, Oxytocin, Prelimbic cortex, Autism spectrum disorder

## Abstract

**Background:**

Dysregulation of the oxytocin (OXT) system is implicated in the pathophysiology of several neuropsychiatric and neurological disorders, particularly autism spectrum disorder (ASD). Our previous study using the *Coiled-coil and C2 domain containing 1a* (*Cc2d1a*) conditional knockout (cKO) mouse model of ASD showed that restoring OXT levels effectively ameliorates irritability-like behavior; however, the mechanisms by which loss of *Cc2d1a* in forebrain excitatory neurons leads to decreased OXT expression in the paraventricular nucleus of the hypothalamus (PVN) remain elusive.

**Methods:**

We used the bottle-brush test (BBT) to assess irritability-like behavior in wild-type (WT) and *Cc2d1a* cKO mice. Retrograde and anterograde trans-synaptic viral tracing were used to map associated neural circuits, and chemogenetic methods were employed to modify neuronal activity. Fiber photometry monitored OXT dynamics in situ, and whole-cell voltage-clamp recordings in ex vivo brain slices assessed synaptic transmission onto PVN OXT neurons.

**Results:**

We found that decreased OXT expression in both magnocellular and parvocellular PVN neurons is evident in adult male *Cc2d1a* cKO mice, without corresponding changes in mRNA levels. Chronic silencing of PVN OXT neurons during adolescence leads to fewer OXT-immunoreactive neurons and heightened irritability-like behavior in adult male WT mice. Using both retrograde and anterograde trans-synaptic viral tracing techniques, we identified that the prelimbic cortex (PrL) indirectly regulates PVN OXT neuronal activity. In male *Cc2d1a* cKO mice, there was a preferential decrease in excitatory synaptic transmission onto PVN OXT neurons. In vivo real-time measurements of OXT dynamics in the posteroventral medial amygdala revealed reduced OXT release during the BBT in male *Cc2d1a* cKO mice. Chronic chemogenetic silencing of the PrL-PVN pathway during adolescence reduces irritability-like behavior in adult male *Cc2d1a* cKO mice.

**Conclusions:**

Our study unveils how forebrain *Cc2d1a* loss reduces OXT expression in the PVN at both molecular and circuit levels, underscoring the critical role of the OXT system as a potential therapeutic target for managing irritability in individuals with ASD.

## Background

Autism spectrum disorder (ASD) is a group of complex neurodevelopmental disorders with highly variable clinical manifestations [1, 2]. Beyond the core phenotypic traits of ASD, such as social communication difficulties and restricted interests or repetitive behaviors, irritability is a common and early-presenting comorbid symptom among autistic individuals, occurring in 20–43% of children with ASD [3, 4]. Irritability in autism manifests through behaviors such as aggression, self-harm, intense tantrums, emotional meltdowns, and withdrawal. It has been linked to increased internalizing and attention problems [5], higher parental stress [6], and reduced adaptive functioning [7, 8]. However, the mechanisms connecting ASD to increased irritability remain unclear, which hampers the development of effective treatment strategies.

The hypothalamic neuropeptide oxytocin (OXT) plays a positive regulatory role in a wide range of social and emotional processes [9, 10]. Impairment of the OXT system is associated with ASD-related deficits in social behavior and cognitive functioning, and OXT treatments have shown beneficial effects on these deficits in children with ASD [11–17]. We previously demonstrated that targeted deletion of *Coiled-coil and C2 domain containing 1a* (*Cc2d1a*) gene, which encodes a scaffold protein that directs signals to multiple intracellular pathways critical for neuronal function, in forebrain excitatory neurons results in increased irritability-like behavior in mice [18]. Moreover, a prominent feature of the *Cc2d1a* conditional knockout (cKO) mouse is a reduction in the number of OXT-immunoreactive neurons in the paraventricular nucleus (PVN) of the hypothalamus, and both exogenous and evoked OXT effectively rescue abnormal irritability-like behavior in these mice, further highlighting the crucial role of OXT in improving ASD symptoms. However, the mechanisms by which loss of *Cc2d1a* in forebrain excitatory neurons causes decreased OXT expression in the PVN remain unexplored.

Given a compelling association between low OXT levels and ASD-related deficits [11–17], this study aimed to clarify the mechanisms underlying the disruption of the PVN OXT system in *Cc2d1a* cKO mice. We show that, consistent with our earlier findings [18], male *Cc2d1a* cKO mice have reduced OXT expression in both magnocellular and parvocellular neurons of the PVN. Using the bottle-brush test (BBT), we find that chronic silencing of PVN OXT neurons during adolescence leads to reduced OXT-immunoreactive neuron number and increased irritability-like behavior in adulthood. OXT dynamics in the posteroventral medial amygdala (MeApv) show decreased OXT release during the BBT in male *Cc2d1a* cKO mice. We identify the prelimbic cortex (PrL) as a critical regulator of PVN OXT neuron activity, acting indirectly via GABAergic interneurons. Chronic chemogenetic silencing of the PrL-PVN pathway during adolescence reduces irritability-like behavior in adult male *Cc2d1a* cKO mice. Surprisingly, female *Cc2d1a* cKO mice show increased irritability-like behavior after ovariectomy, correlating with decreased arginine vasopressin (AVP) but not OXT expression in the PVN. These findings represent a significant advance in understanding the mechanisms underlying ASD-related irritability-like behavior and highlight the therapeutic potential of OXT in clinical practice.

## Materials and methods

### Animals

Male and female mice aged 2–24 weeks were used. We used the following mouse lines: *Emx1*-Cre mice (The Jackson Laboratory, Strain #: 005628) and *Cc2d1*a cKO mice, which were generated by crossing *Cc2d1a*-floxed (*Cc2d1a*^f/f^) mice with *Emx1*-Cre mice; *Oxt*-Cre::hM4D(Gi) mice, which were generated by crossing R26-LSL-Gi-DREADD mice (The Jackson Laboratory, Strain #: 026219) with *Oxt*-Ires-Cre mice (The Jackson Laboratory, Strain #: 024234) and Ai14 mice (The Jackson Laboratory, Strain #: 007914). The *Cc2d1a*^f/f^ mouse line with Cre-dependent excision of exons 12–14 was generated, genotyped, and maintained as previously described [19, 20]. *Cc2d1a*^f/f^ mice were used as the wild-type (WT) littermates for comparison with the homozygous *Cc2d1a* cKO mice. *Cc2d1a* cKO mice were genotyped using a polymerase chain reaction (PCR)-based method using genomic DNA extracted from tail samples. The following primer sequences were used: *Cc2d1a*, forward (5’-AGCACTGTTTGCGTCAGGGATACT-3’) and reverse (5’-TCTATACCGAAGATGGAGCCTGGG-3’); *Oxt*-Cre, forward mutant (5’-ACACCGGCCTTATTCCAAG-3’), forward WT (5’-AGCCTGCTGGACTGTTTTTG-3’) and reverse (5’-TTTGCAGCTCAGAACACTGAC-3’); Cre, forward (5’-AAGAACCTGATGGACATGTTCAGGGATCG-3’) and reverse (5’-CCACCGTCAGTACGTGAGATATCTTTA ACC-3’). Mice were housed in groups of 3–4 per cage in a temperature-controlled (22 ± 1 °C) and humidity-controlled (40–60%) environment with a reverse 12 h light/dark cycle (lights on at 6:00 am) and ad libitum access to water and regular chow (13.5% kcal from fat, Laboratory Autoclavable Rodent Diet 5010, LabDiet, St. Louis, MO). All experimental protocols were approved by the Institutional Animal Care and Use Committee (IACUC) at National Cheng Kung University (Approval No. 113038), which adheres to the NIH Guidelines for the Ethical Treatment of Animals. Animals were used in accordance with the “3Rs” principles (Replacement, Reduction, and Refinement) throughout all experimental procedures. A total of 207 mice were used in this study. The investigators were blinded to the treatment groups during the experiments and outcome assessments.

### Ovariectomy

The ovariectomy procedure was performed as previously described [22]. Female mice underwent surgical ovariectomy (OVX) at 3 weeks old. OVX was performed through small bilateral dorsal flank incisions under anesthesia with alfaxalone (50 mg/kg) and dexmedetomidine (0.5 mg/kg), using aseptic technique. The skin on the lower back, just above the hips, was shaved and sterilized. A small dorsolateral incision was made, and the ovaries were removed. Mice in the sham group underwent the same procedure, but the ovaries remained intact afterward.

### Recombinant adeno-associated virus (AAV) vector production

DNA plasmids, including pAAV-hSyn-DIO-hM4D(Gi)-mCherry (plasmid #44362), pAAV-hSyn-DIO-mCherry (plasmid #50459), pAAV-hSyn-GRAB_OT1.0 (plasmid #185386), pAAV-hSyn-Cre (plasmid #105553), and pAAV-hSyn-DIO-EGFP (plasmid #50457), were purchased from Addgene. Plasmid DNA was amplified, purified, and collected using the QIAGEN Plasmid Maxi Prep Kit according to the manufacturer’s instructions. The purified plasmid was combined with a CaCl_2_ solution containing the plasmid encoding the AAV capsid and co-transfected into HEK293GP cells using the calcium phosphate co-precipitation method, as previously described [21]. Transfected cells were harvested 72 h after transfection, and the viruses were purified with the AAV Purification Mega Kit (Cell Biolabs Inc.). The virus titer of the preparation was 7.2 × 10^12^ particles/mL and stored in aliquots at −80 °C until use.

### Stereotaxic viral injections and chemogenetic manipulations

Stereotaxic viral injections and chemogenetic manipulations were performed as previously described [23]. All experimental animals were anesthetized with a mixture of 50 mg/kg alfaxalone (Alfaxan, Zoetis) and 0.5 mg/kg dexmedetomidine (Dexdomitor, Vetnostrum). For chronic chemogenetic silencing of PrL glutamatergic neurons, *Cc2d1a* cKO mice were bilaterally injected with AAV_DJ_-hSyn-DIO-hM4D(Gi)-mCherry (0.5μL at 0.1 μL/min) into the PrL [+ 1.9 mm anteroposterior (AP), ± 0.3 mm mediolateral (ML), −2.1 mm dorsoventral (DV)] by using a 1 μL Hamilton syringe with a 34-gauge blunt tip needle. One week after viral injections, mice were treated with water containing either a vehicle [0.1% dimethyl sulfoxide (DMSO) in saline (0.9%)] or clozapine-*N*-oxide (CNO, 5 mg/200 mL in 0.1% DMSO in saline, Cat#4936, Tocris Bioscience) for 2 weeks.

### Retrograde labeling of PVN magnocellular OXT neurons

To retrogradely label PVN magnocellular OXT neurons, we used a modified version of an established protocol [24]. WT and *Cc2d1a* cKO mice were injected intraperitoneally with 15 mg/kg of Fluoro-Gold (FG). One week after mice were anesthetized with a combination of 50 mg/kg alfaxalone and 0.5 mg/kg dexmedetomidine, their brains were harvested. To quantify PVN magnocellular OXT neurons, cells co-expressing OXT and FG (OXT^+^FG^+^) were counted as magnocellular OXT neurons.

### Fiber photometry detection of OXT signals in situ

A commercial fiber photometry system (RWD Life Science) was used to record OXT signals in vivo from behaving animals. AAV_9_-hSyn-GRAB_OT1.0 was bilaterally infused into the MeApv of mice, and subsequently, an optic fiber (diameter, 400 μm, NA, 0.5) was implanted 100 μm above the virus injection site (−1.5 mm AP, ± 2.5 mm ML, −5.6 mm DV from Bregma). We secured the fiber optic to the skull using dental cement. The mice underwent BBT 2 weeks after viral injection. We used a blue LED to deliver excitation light at 470 nm and collect OT1.0 emission at 30 Hz. Change in fluorescence (ΔF/F) was calculated as (F-F0)/F0, where F0 stands for the baseline fluorescence signal. The OXT activity was also estimated using the area under the curve (AUC) of the signal, computed as the integral of the ΔF/F trace over time. The OXT signals for each trial were synchronized with video-annotated behavioral events.

### Behavioral test

All behavioral tests were conducted during the dark phase and performed in a genotype-blind manner. Mice were acclimated to the testing room for at least 1 h before testing. Male and female mice, aged 12–15 weeks, were used in the experiments to reduce variability caused by changes during adolescence. All behavioral experiments were videotaped for later offline analysis. Well-trained observers were blinded to the experimental groups when scoring behavioral responses in randomized animals. The mean of the measurements for each mouse response was used for data analysis.

### Irritability-like behavior

To measure irritability-like behavior, the BBT was adapted from established protocols [18, 25, 26]. In brief, the BBT consisted of 10 trials with 10-s intertrial intervals per mouse, performed in a clean plastic cage (37.5 cm × 17 cm × 18 cm) with fresh bedding. At the start of each session, the mouse began at the back of the cage. Each session consisted of five steps, in order. Step 1: The brush spun rapidly toward the mouse for about 2 s. Step 2: The brush was rotated around the mouse’s whiskers for about 2 s. Step 3: The brush was quickly rotating away from the mouse towards the front of the cage for about 2 s. Step 4: The brush was rotated at the starting point for about 2 s. Step 5: The brush was hung vertically at the starting point for about 10 s before being completely removed from the cage. Both aggressive responses (exploring the brush, following the brush, and tail-rattling) and defensive responses (escaping from the brush, digging, jumping, rearing, and grooming) were recorded in all trials. We chose these responses for analysis because they are clearly distinguishable behaviors that can be categorized as approach (aggressive) and avoidance (defensive) [25, 26].

### Immunohistochemistry

Immunofluorescence staining was carried out as previously described [18]. Mice were deeply anesthetized with a mixture of 50 mg/kg alfaxalone and 0.5 mg/kg dexmedetomidine, then transcardially perfused with 4 °C phosphate-buffered saline (PBS), followed by 4% paraformaldehyde (PFA) in 0.1 M PBS, pH 7.4. After perfusion, brains were removed and fixed in 4% PFA for 24 h at 4 °C, then transferred to a solution containing 30% sucrose, which was maintained at 4 °C for at least 48 h before slicing. Coronal slices were cut to a thickness of 20 μm, washed with 0.4% Triton X-100, and then incubated in blocking buffer containing 3% bovine serum albumin (BSA, Merck, Cat#A7906) and 0.3% Triton X-100 for at least 1 h. After blocking, brain slices were incubated with primary antibody against glutamic acid decarboxylase 67 (GAD67, 1:500, Sigma-Aldrich, Cat#G5419; RRID: AB_261978) or OXT (1:2000, Millipore, Cat#MAB5296; RRID: AB_11212999; 1:2000 or Millipore, Cat#AB911; RRID: AB_2157629) for 24 h at 4 °C in blocking solution. Finally, sections were washed with PBS-T (0.4% Triton X-100 in PBS) and then incubated with secondary Alexa-Fluor conjugated 488 (1:1000, Invitrogen, Cat#A11001; RRID: AB_2534069), 568 (1:1000, Invitrogen, Cat#A11004; RRID: AB_2534072) and 405 antibodies (1:1000, Invitrogen, Cat#A31556; RRID: AB_221605) for 1 h at room temperature. The immunostained brain slices were placed on separate gelatin-coated glass slides, rinsed thoroughly with PBS, and mounted with either Mounting Medium containing 4',6-diamidino-2-phenylindole (DAPI) (Abcam, Cat#ab104139) or Fluoromount-G® (SouthernBiotech, Cat#0100–01). Post hoc tissue images were captured using an Olympus FluoView FV3000 confocal laser scanning microscope. To quantify OXT^+^, FG^+^, and GFP^+^ cells, 6–7 slices from each mouse were counted bilaterally. All images were automatically analyzed using the built-in ImageJ function (RRID: SCR_001935). For colocalization analysis of OXT^+^GFP^+^ or OXT^+^FG^+^ cells, co-expressing cells with OXT and GFP or FG fluorescent signals were counted.

### Slice preparation and electrophysiological recordings

Acute coronal slices containing the PVN were prepared from 12 to 15-week-old male WT or *Cc2d1a* cKO mice as previously described [27]. Briefly, mice were decapitated under isoflurane anesthesia, and brains were rapidly removed and immersed in ice-cold oxygenated sucrose cutting solution containing (in mM): 234 sucrose, 2.5 KCl, 0.5 CaCl_2_, 7 MgSO_4_, 25 NaHCO_3_, 1.25 NaH_2_PO_4,_ and 20 glucose at pH 7.3–7.4 and equilibrated with 95% O_2_−5% CO_2_. Slices (250 μm) were prepared using a vibrating microtome (VT1200S; Leica) and transferred to a home-made submersion-type holding chamber of artificial cerebrospinal fluid (aCSF) containing (in mM): 117 NaCl, 4.7 KCl, 2.5 CaCl_2_, 1.2 MgCl_2_, 25 NaHCO_3_, 1.2 NaH_2_PO_4_ and 11 glucose at pH 7.3–7.4 and equilibrated with 95% O_2_−5% CO_2_ and kept at room temperature for at least 1 h before recordings.

For recordings, one slice was transferred to the submersion-type recording chamber and secured to the glass bottom using a nylon grid on a platinum frame. The chamber was constantly perfused with aCSF at 32.0 ± 0.5 °C with a rate of 2–3 mL/minute. Slices were visualized using a 60 × (1.0 NA) water-immersion objective lens under infrared differential interference contrast imaging on an Olympus BX51W1 upright microscope. Conventional whole-cell patch-clamp recordings were performed on PVN OXT-expressing neurons using an Axopatch 200B amplifier (Molecular Devices; RRID: SCR_018866). Data acquisition and analysis were performed using a digitizer (Digidata 1440 A, Molecular Devices; RRID: SCR_021038) and the pCLAMP 9 software (Molecular Devices; RRID: SCR_011323). The composition of the internal solution was (in mM): Cs^+^-based internal solution was used (in mM): 110 Cs-gluconate, 10 CsCl_2_, 1 EGTA, 1 CaCl_2_, 10 HEPES, 1 Mg-ATP, 1 QX-314, and 0.5% (w/v) biocytin (pH 7.3 adjusted with CsOH). Biocytin was routinely added to the internal solution to fill the recorded neurons for post hoc cell identification. For recording miniature inhibitory postsynaptic currents (mIPSCs) and miniature excitatory postsynaptic currents (mEPSCs), PVN OXT-expressing neurons were held in voltage-clamp mode at a holding potential of 0 mV or −60 mV, respectively, with tetrodotoxin (TTX, 1 μM) added to the bath. mIPSCs were recorded in the presence of 6-cyano-7-nitroquinoxaline-2,3-dione (CNQX, 10 μM) and 2-amino-5-phosphonovaleric acid (APV, 50 μM), and mEPSCs were recorded in the presence of gabazine (10 μM). Data was analyzed offline using commercially available software (Mini Analysis 4.3; Synaptosoft, RRID: SCR_002184) [28]. Means were calculated from 5-min epochs recorded. The analysis threshold was set to 3 times the root-mean-square of the background noise, and each event was also confirmed through visual inspection after detection.

### RNA extraction and quantitative polymerase chain reaction (qPCR) analysis

Mice were anesthetized with a mixture of 50 mg/kg alfaxalone and 0.5 mg/kg dexmedetomidine, and their brains were quickly removed and immersed in cold, oxygenated PBS. The PVN-containing brain slices were prepared with a vibrating microtome (VT1200S; Leica) and then dissected. Brain tissue was stored in liquid nitrogen before RNA extraction. Total RNA was isolated using TRIzol reagent (Invitrogen) and subsequently reverse-transcribed into cDNA with the cDNA Reverse Transcription Kit (Applied Biosystems). After cDNA synthesis, gene-specific primers were designed using NCBI Primer Design. The cDNA concentration was measured with the NanoDrop 1000 (Thermo Fisher). Real-time qPCR was performed in triplicate using an SYBR Green MasterMix (Invitrogen; Cat# K0221) on StepOne Real-Time PCR Systems (Applied Biosystems). Relative gene expression was determined using the double delta Ct (ΔΔCt) method. Gene expression levels were normalized to the housekeeping gene *GAPDH*. Primer sets for qPCR were the following: *GAPDH*, forward (5’-CCATCACCATCTTCCAGGAG-3’) and reverse (5’-CCTGCTTCACCACCTTCTTG-3’); *Oxt*, forward (5’-CCATCACCTACAGCGGATCT-3’) and reverse (5’-GGGAGACACTTGCGCATATC-3’).

### Statistical analysis

Sample sizes were determined based on prior work from our laboratory [18, 20] and calculated using a power analysis (two-tailed, with a significance level of 0.05 and 80% power) in G*Power software. No specific randomization method was applied. Animals were randomly assigned to experimental groups. Data are presented as the mean ± SEM and analyzed by the GraphPad Prism 6 software (GraphPad Software Inc., RRID: SCR_002798). The D'Agostino & Pearson normality test was used to analyze the normality of data distribution. The Mann–Whitney *U*-test was used for within-group comparison. The differences among multiple groups were assessed using a two-way ANOVA followed by Tukey’s post hoc multiple-comparison test. The number of animals used is indicated by *N*. Differences were considered significant at *P* < 0.05.

## Results

### Male *Cc2d1a* cKO mice show decreased OXT expression in the PVN

**Fig. 1:**
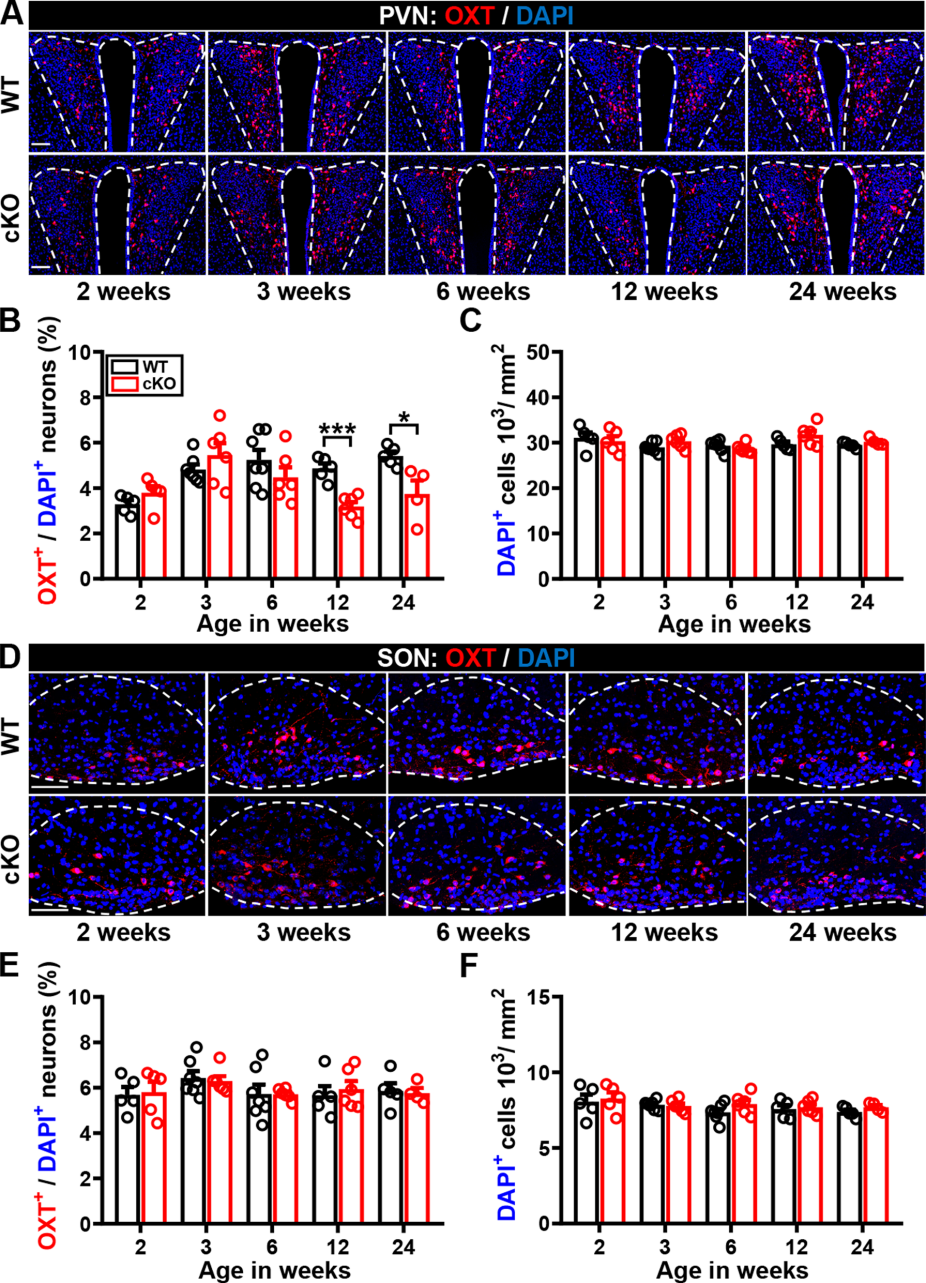
Male *Cc2d1a* cKO mice exhibited fewer OXT-expressing neurons in the PVN at the adult stage.** A** Representative images showing OXT immunoreactivity in the PVN of male WT and *Cc2d1a* cKO mice during development and adulthood. Scale bar, 100 μm. **B**, **C** Quantification of total number of DAPI^+^ cells (mouse numbers: *N*_WT-2 week_ = 5, *N*_cKO-2 week_ = 5; Mann–Whitney *U*-test, *P* = 0.841; *N*_WT-3 week_ = 7, *N*_cKO-3 week_ = 6, *P* = 0.1375; *N*_WT-6 week_ = 7, *N*_cKO-6 week_ = 6, *P* = 0.2949; *N*_WT-12 week_ = 5, *N*_cKO-12 week_ = 6, *P* = 0.1255; *N*_WT-24 week_ = 5, *N*_cKO-24 week_ = 4, *P* = 0.190; **B**) and the percentage of OXT^+^/DAPI^+^ neurons (mouse numbers: *N*_WT-2 week_ = 5, *N*_cKO-2 week_ = 5; Mann–Whitney *U*-test, *P* = 0.150; *N*_WT-3 week_ = 7, *N*_cKO-3 week_ = 6, *P* = 0.4452; *N*_WT-6 week_ = 7, *N*_cKO-6 week_ = 6, *P* = 0.2949; *N*_WT-12 week_ = 5, *N*_cKO-12 week_ = 6, *P* = 0.0043; *N*_WT-24 week_ = 5, *N*_cKO-24 week_ = 4, *P* = 0.0159; **C**) in the PVN of male WT and *Cc2d1a* cKO mice during development and adulthood. **D** Representative images showing OXT immunoreactivity in the SON of male WT and *Cc2d1a* cKO mice during development and adulthood. Scale bar, 100 μm. **E**, **F** Quantification of total number of DAPI^+^ cells (mouse numbers: *N*_WT-2 week_ = 5, *N*_cKO-2 week_ = 5; Mann–Whitney *U*-test, *P* = 0.6905; *N*_WT-3 week_ = 7, *N*_cKO-3 week_ = 6, *P* = 0.7308; *N*_WT-6 week_ = 7, *N*_cKO-6 week_ = 6, *P* = 0.2949; *N*_WT-12 week_ = 5, *N*_cKO-12 week_ = 6, *P* = 0.6623; *N*_WT-24 week_ = 5, *N*_cKO-24 week_ = 4, *P* = 0.1905; **E**) and the percentage of OXT^+^/DAPI^+^ neurons (mouse numbers: *N*_WT-2 week_ = 5, *N*_cKO-2 week_ = 5; Mann–Whitney *U*-test, *P* = 0.8413; *N*_WT-3 week_ = 7, *N*_cKO-3 week_ = 6, *P* > 0.9999; *N*_WT-6 week_ = 7, *N*_cKO-6 week_ = 6, *P* = 0.6282; *N*_WT-12 week_ = 5, *N*_cKO-12 week_ =, *P* = 0.9307; *N*_WT-24 week_ = 5, *N*_cKO-24 week_ = 4, *P* = 0.7302; **F**) in the SON of male WT and *Cc2d1a* cKO mice during development and adulthood. Data are presented as mean ± SEM. *P < 0.05 and **P < 0.01 as compared with the WT group

Building on our previous work showing a decrease in the number of OXT-immunoreactive neurons in the PVN of male *Cc2d1a* cKO mice [18], we first examined the developmental stage at which this reduction begins. Immunohistochemical analysis confirmed that OXT-expressing neurons are distributed throughout the rostral-to-caudal extent of the PVN (Fig. 1A) and the supraoptic nucleus (SON) of the hypothalamus (Fig. 1D) in both developing and adult mouse brains. We observed a lower percentage of OXT-expressing neurons in the PVN (Fig. 1B) but not in the SON (Fig. 1E) of male *Cc2d1a* cKO mice compared with WT mice at 12 and 24 weeks of age, whereas total cell numbers in both regions did not differ across groups (Fig. 1C, F).

**Fig. 2:**
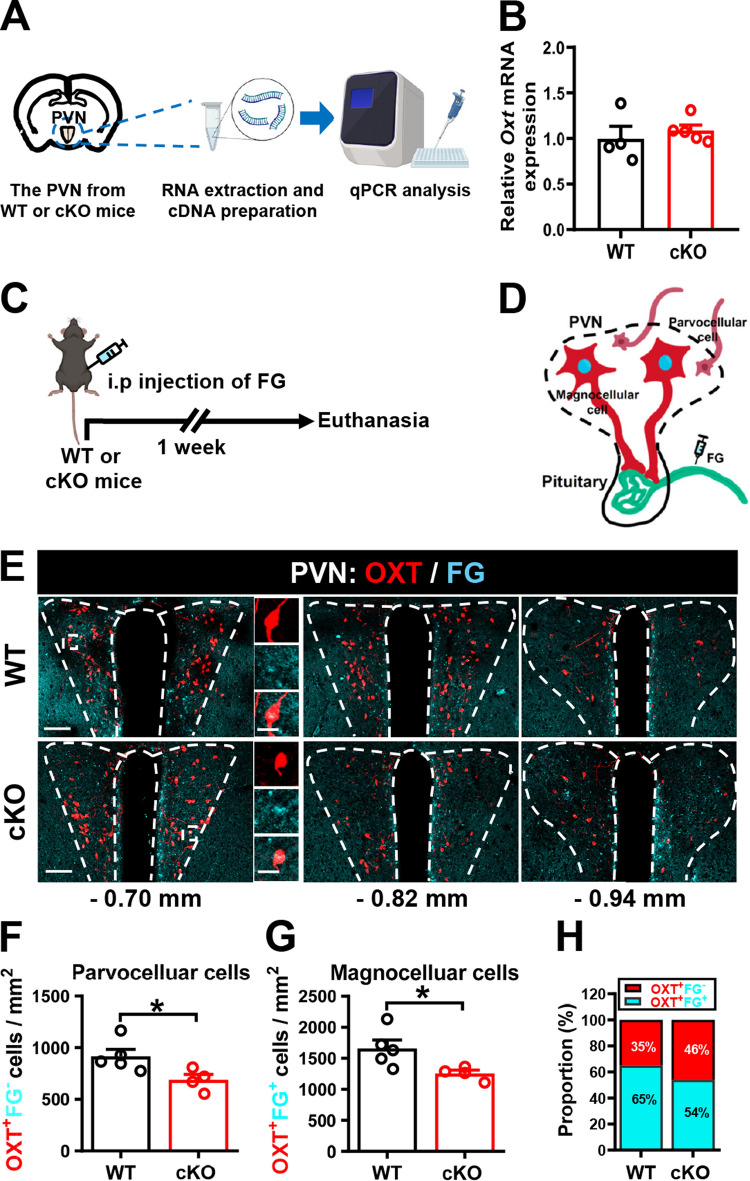
Male *Cc2d1a* cKO mice show decreased OXT expression in magnocellular and parvocellular neurons of the PVN.** A** Schematic diagram of the experimental design. PVN-containing brain slices were collected from 12-week-old male WT and *Cc2d1a* cKO mice, and the extracted RNA was analyzed by qPCR analysis. **B** Bar graphs with dots showing the relative *Oxt* mRNA expression, normalized to *GAPDH*, in the PVN of WT and *Cc2d1a* cKO mice (mouse number: *N*_WT_ = 4, *N*_cKO_ = 5; Mann-Whitney *U*-test, *P* = 0.2857). **C** Schematic diagram of the experimental timeline and design. One week after intraperitoneal FG injection, WT and *Cc2d1a* cKO mice were euthanized, and their brains were collected for immunohistochemical analysis. **D** Graphic illustration of FG retrograde labeling of magnocellular OXT-expressing neurons in the PVN. **E** Representative image showing OXT and FG expression at different anteroposterior levels of the PVN in male WT and *Cc2d1a* cKO mice. Scale bar: 100 μm. Magnified images of the rectangle show OXT^+^FG^+^ cells. Scale bar, 20 µm. **F** Quantification of the number of OXT^+^FG^-^/mm^2^ cells (parvocellular OXT-expressing neurons) in the PVN of male WT and *Cc2d1a* cKO mice (mouse numbers: *N*_WT_ = 5, *N*_cKO_ = 4; Mann-Whitney *U*-test, *P* = 0.0317). **G** Quantification of the number of OXT^+^FG^+^/mm^2^ cells (magnocellular OXT-expressing neurons) in the PVN of male WT and *Cc2d1a* cKO mice (mouse numbers: *N*_WT_ = 5, *N*_cKO_ = 4; Mann-Whitney *U*-test, *P* = 0.0317). **H** Comparison of the proportions of parvocellular and magnocellular OXT-expressing neurons in the PVN of male WT and *Cc2d1a* cKO mice (mouse numbers: *N*_WT_ = 5, *N*_cKO_ = 4; Chi-square test; *P* = 0.1131). Data are presented as mean ± SEM. *P < 0.05 as compared with the WT group. **A** and **C** were created with BioRender.com.

We next examined whether the decrease in OXT-expressing neurons in the PVN of male *Cc2d1a* cKO mice results from altered *Oxt* transcript levels by quantifying Oxt mRNA levels using qPCR (Fig. 2A). There was no significant difference in *Oxt* mRNA levels in the PVN between male *Cc2d1a* cKO and WT mice at 12 weeks of age (Fig. 2B). Because PVN OXT neurons are traditionally classified into magnocellular and parvocellular subtypes based on their size, shape, and projections [29], we sought to identify which PVN OXT neuron subtypes are affected in male *Cc2d1a* cKO mice. We intraperitoneally injected the retrograde tracer, FG (Fig. 2C), to specifically label magnocellular neurons but not parvocellular neurons because only magnocellular OXT neurons project directly to the posterior pituitary, where they release OXT into the systemic blood circulation to act on peripheral target organs [30] (Fig. 2D). Consistent with previous studies [13, 24], double labeling of OXT and FG shows that FG^+^ magnocellular OXT neurons are primarily distributed in the anterior part of the PVN, whereas FG^−^ parvocellular OXT neurons are found in the posterior part of the PVN (Fig. 2E). Notably, the numbers of both FG^+^ magnocellular (Fig. 2F) and FG^−^ parvocellular OXT neurons (Fig. 2G) were lower in male *Cc2d1a* cKO mice than in WT mice. There were no significant differences between male *Cc2d1a* cKO and WT mice in the proportions of FG^+^ magnocellular and FG^−^ parvocellular OXT neurons in the PVN (Fig. 2H).

### Activity-dependent regulation of OXT expression in the PVN

**Fig. 3:**
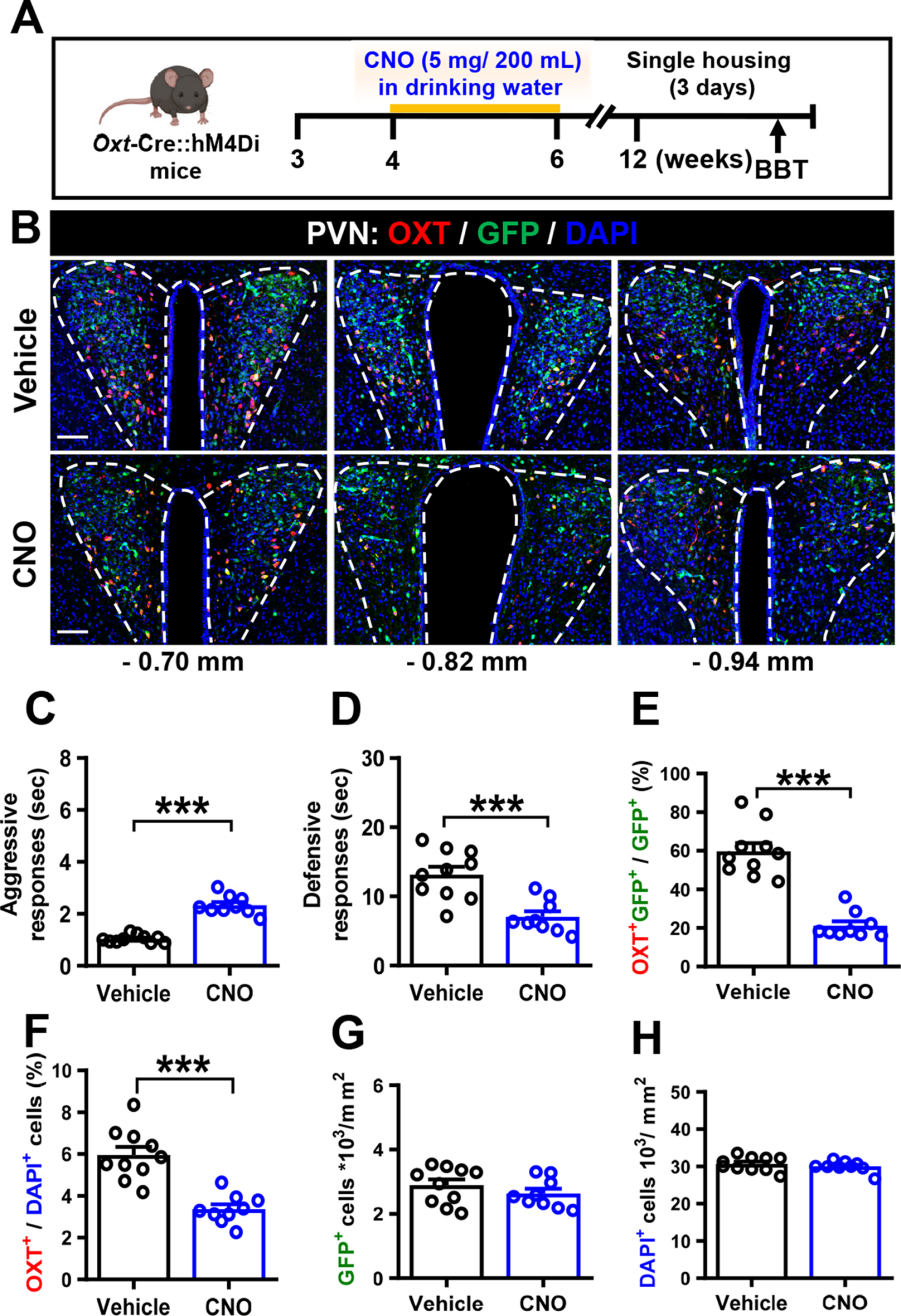
Inhibition of OXT-expressing neuronal activity results in fewer OXT-expressing neurons in the PVN and increased irritability-like behavior. **A** Schematic diagram of the experimental timeline and design. One week after weaning, male *Oxt*-Cre::hM4Di mice received vehicle or CNO in their drinking water for 2 weeks, then underwent the BBT and were housed individually for 3 days. **B** Representative image showing OXT and GFP expression in the PVN of male *Oxt*-Cre::hM4Di mice treated with vehicle or CNO. Scale bar, 100 μm. **C** Comparison of time spent by male *Oxt*-Cre::hM4Di mice treated with vehicle or CNO during aggressive responses to the BBT (mouse numbers: *N*_Vehicle_ = 10, *N*_CNO_ = 9; Mann–Whitney *U*-test, *U* = 0, *P* < 0.0001). **D** Comparison of time spent by male *Oxt*-Cre::hM4Di mice treated with vehicle or CNO during defensive responses to the BBT (mouse numbers: *N*_Vehicle_ = 10, *N*_CNO_ = 9; Mann–Whitney *U*-test, *P* = 0.001). **E**, **F** Quantification of the percentage of OXT^+^GFP^+^/GFP^+^ neurons (mouse numbers: *N*_Vehicle_ = 10, *N*_CNO_ = 9 Mann–Whitney *U*-test, *P* < 0.0001; **E**) and OXT^+^/DAPI^+^ neurons (mouse numbers: *N*_Vehicle_ = 10, *N*_CNO_ = 9; Mann–Whitney *U*-test, *P* < 0.0001; **F**) in the PVN of male *Oxt*-Cre::hM4Di mice treated with vehicle or CNO. **G**, **H** Quantification of total number of GFP^+^ cells (mouse numbers: *N*_Vehicle_ = 10, *N*_CNO_ = 9; Mann–Whitney *U*-test, *P* = 0.3562; **G**) and DAPI^+^ cells (mouse numbers: *N*_Vehicle_ = 10, *N*_CNO_ = 9; Mann–Whitney *U*-test, *P* = 0.6607; **H**) in the PVN of male *Oxt*-Cre::hM4Di mice treated with vehicle or CNO. Data are presented as mean ± SEM. ***P < 0.0001 as compared with the vehicle group

To investigate whether neuronal activity causally affects OXT expression, we reduced PVN OXT neuron activity using a chemogenetic approach based on designer receptors exclusively activated by designer drugs (DREADDs). Male *Oxt*-Cre::hM4D(Gi) mice were given drinking water containing either vehicle or CNO (5 mg/200 mL) for 2 weeks. At 12 weeks of age, after 3 days of individual housing, their aggressive and defensive responses to the BBT were examined (Fig. 3A). We verified the neuronal specificity of viral expression by imaging GFP, which was confined to OXT-immunoreactive neurons in the PVN (Fig. 3B). We found that CNO-treated male *Oxt*-Cre::hM4D(Gi) mice exhibited increased aggressive responses (Fig. 3C) and reduced defensive responses (Fig. 3D) to the BBT compared with vehicle-treated male *Oxt*-Cre::hM4D(Gi) mice. Consistently, we observed a significant decrease in the percentages of OXT^+^/GFP^+^ (Fig. 3E) and OXT^+^/DAPI^+^ (Fig. 3F) cells in the PVN of CNO-treated male *Oxt*-Cre::hM4D(Gi) mice compared with vehicle-treated male *Oxt*-Cre::hM4D(Gi) mice, whereas the total cell numbers of GFP^+^ cells (Fig. 3G) and DAPI^+^ cells (Fig. 3H) did not differ between groups.

**Fig. 4:**
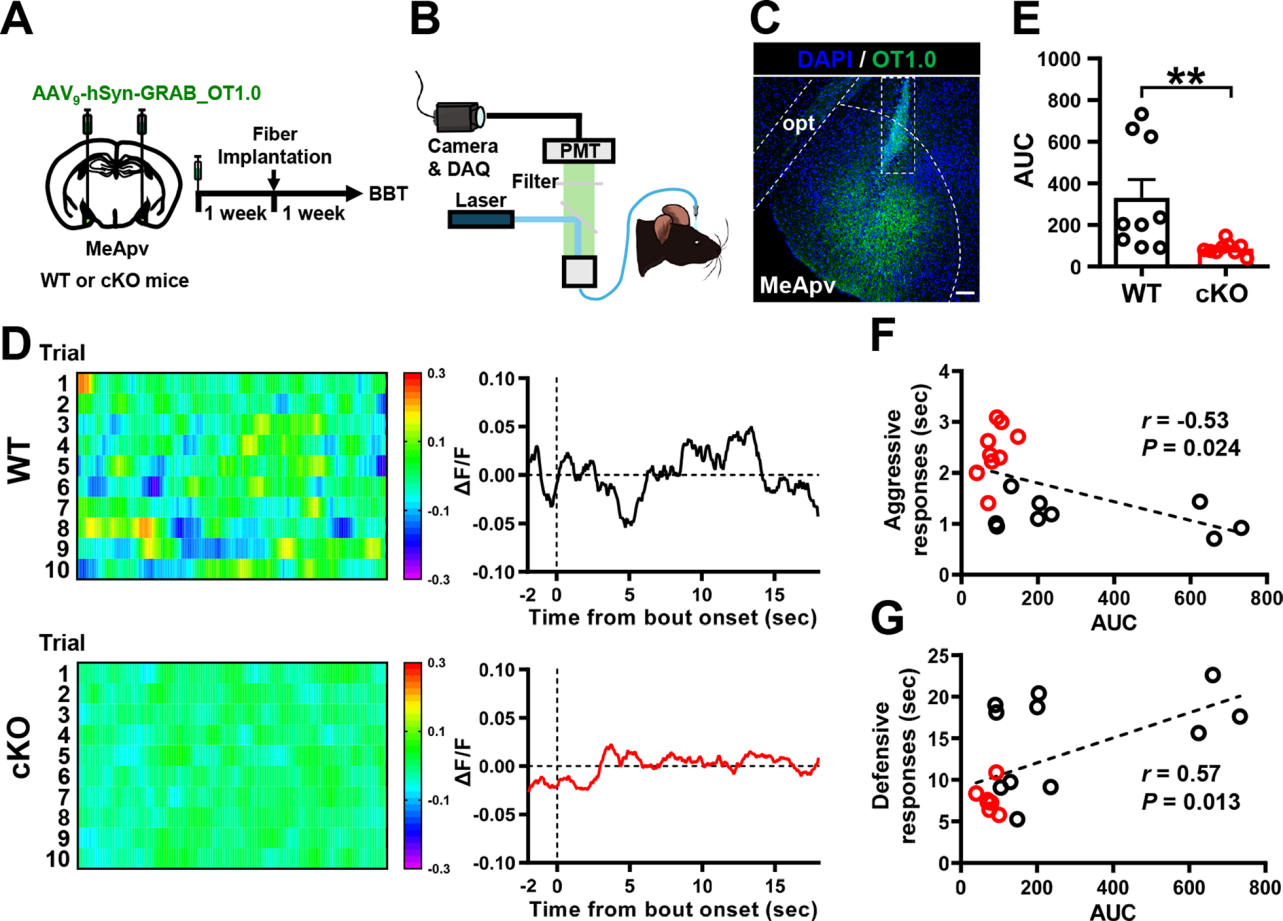
Male *Cc2d1a* cKO mice exhibited reduced OXT release in the MeApv during the BBT.** A** Schematic diagram of the experimental design. AAV_9_-hSyn-BRAB_OT1.0 was bilaterally injected into the MeApv of male WT and *Cc2d1a* cKO mice, one week after implantation of an optical fiber above the injection site. One week after optic fiber implantation, mice were housed individually for 3 days, then their OXT release fluorescent signal in the MeApv was recorded while they participated in the BBT. **B** Graphical illustration of the OXT fluorescent signal during the test phase, recorded by the photometry system. **C** Representative image showing the expression of OT1.0 in the MeApv of a mouse, with the optic fiber placement indicated. Scale bar, 100 μm. **D** Representative heat maps (left) and average ΔF/F traces (right) showing OXT release activity in one example male WT mouse and one example male *Cc2d1a* cKO mouse, aligned to the onset of the bottle brush introduced into the testing cage. The trials were obtained from the same WT mouse and *Cc2d1a* cKO mouse. **E** Quantification bar graphs with dots showing the analysis of the area under the curve (AUC) for OT1.0 signals in male WT or *Cc2d1a* cKO mice during the BBT session (mouse numbers: *N*_WT_ = 9, *N*_cKO_ = 9; Mann–Whitney *U*-test, *P* = 0.004). **F** Correlation plot showing the relationship between the detected OT1.0 signal AUC and aggressive responses during the BBT (mouse numbers: *N*_WT_ = 9, *N*_cKO_ = 9; Pearson correlation test, *r* = −0.53, *P* = 0.024). **G** Correlation plot showing the relationship between the detected OT1.0 signal AUC and the defensive responses during the BBT (mouse numbers: *N*_WT_ = 9, *N*_cKO_ = 9; Pearson correlation test, *r* = 0.57, *P* = 0.013). Data are presented as mean ± SEM. **P < 0.01 as compared with the WT group

To directly monitor OXT dynamics in the posteroventral medial amygdala (MeApv) during the BBT in male WT and *Cc2d1a* cKO mice, we targeted the MeApv with AAV_9_-hSyn-GRAB_OT1.0 to express the genetically encoded sensor GRAB_OT1.0, and recorded responses in freely moving mice using fiber photometry. One week after the viral infection, the mice underwent optical fiber implantation and were subjected to the BBT one week later (Fig. 4A). Before recording began, the implanted optical fiber was connected to the photometry system with a jumper cable to deliver excitation light and collect real-time tracking of the OXT signal during the test phase (Fig. 4B). Post hoc histological analysis of AAV_9_-hSyn-GRAB_OT1.0-injected brains confirmed that GRAB_OT1.0 expression is mainly confined to the MeApv, and that the optic fiber was correctly implanted (Fig. 4C). By aligning GRAB_OT1.0 signals with video-scored behavioral actions observed during the BBT, we identified changes in GRAB_OT1.0 signals that differed between aggressive and defensive responses during the BBT (Fig. 4D). We measured total response extent as the AUC and observed a significant decrease in male *Cc2d1a* cKO mice compared with WT mice (Fig. 4E), indicating reduced OXT dynamics during the BBT. Further correlation analysis revealed that the AUC of OXT dynamics during the BBT was negatively correlated with aggressive responses (*r* = −0.53, *P* = 0.024; Fig. 4F) and positively correlated with defensive responses (*r* = 0.57, *P* = 0.013; Fig. 4G), suggesting that OXT secretion is strongly linked to the regulation of irritability-like behavior.

### Male *Cc2d1a* cKO mice show reduced excitatory synaptic transmission onto PVN OXT neurons

**Fig. 5:**
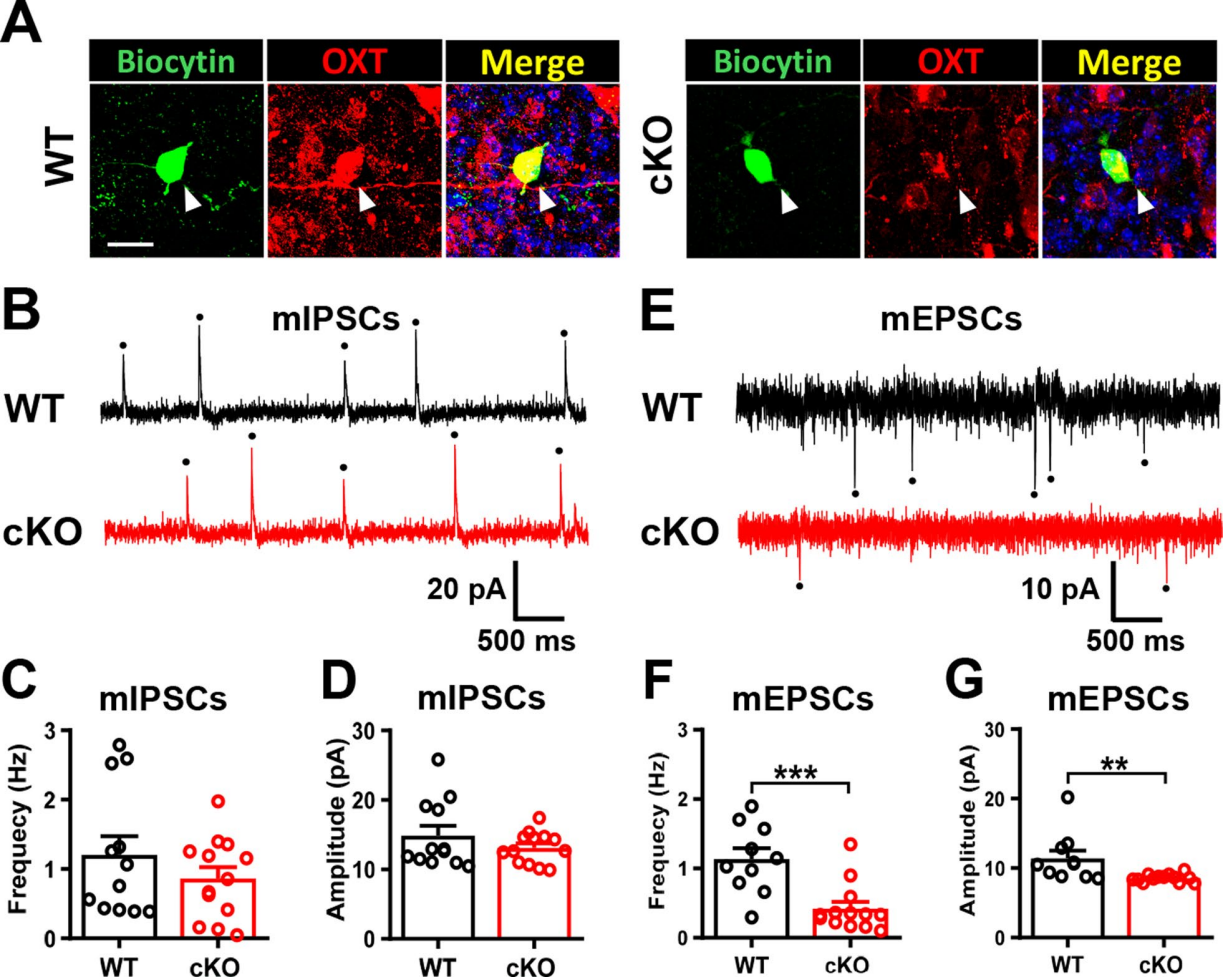
Male *Cc2d1a* cKO mice show decreased excitatory synaptic transmission onto PVN OXT neurons. **A** Representative image showing the recorded OXT-expressing neurons in the PVN of WT (left) and *Cc2d1a* cKO (right) mice. Scale bar, 20 μm. **B** Representative traces of mIPSCs in PVN OXT-expressing neurons from male WT and *Cc2d1a* cKO mice. **C** Comparison of the mean frequency of mIPSCs in PVN OXT-expressing neurons from male WT and *Cc2d1a* cKO mice (WT: *n* = 12 neurons from 4 mice, *Cc2d1a* cKO: *n* = 13 neurons from 4 mice; Mann–Whitney *U*-test, *P* = 0.5743). **D** Comparison of the mean amplitude of mIPSCs in PVN OXT-expressing neurons from male WT and *Cc2d1a* cKO mice (WT: *n* = 12 neurons from 4 mice, *Cc2d1a* cKO: *n* = 13 neurons from 4 mice; Mann–Whitney *U*-test, *P* = 0.6114). **E** Representative traces of mEPSCs in PVN OXT-expressing neurons from male WT and *Cc2d1a* cKO mice. **F** Comparison of the mean frequency of mEPSCs in PVN OXT-expressing neurons from male WT and *Cc2d1a* cKO mice (WT: *n* = 10 neurons from 4 mice, *Cc2d1a* cKO: *n* = 13 neurons from 4 mice; Mann–Whitney *U*-test, *P* = 0.0015). **G** Comparison of the mean amplitude of mEPSCs in PVN OXT-expressing neurons from male WT and *Cc2d1a* cKO mice (WT: *n* = 10 neurons from 4 mice, *Cc2d1a* cKO: *n* = 13 neurons from 4 mice; Mann–Whitney *U*-test, *P* = 0.0011). Data are presented as mean ± SEM. **P < 0.01 and ***P < 0.001 as compared with the WT group

The data above indicate that decreased OXT expression is associated with lower activity of PVN OXT neurons. We next examined whether deletion of *Cc2d1a* in forebrain excitatory neurons alters the balance of excitatory and inhibitory synaptic transmission onto PVN OXT neurons, thereby making them less excitable. To test this, we conducted whole-cell voltage-clamp recordings in acute slices to monitor synaptic currents of PVN OXT neurons. Biocytin labeling followed by OXT immunostaining was used to retrospectively verify the identity of PVN OXT neurons from which recordings were taken (Fig. 5A). We found no significant differences in mean mIPSC frequency and amplitude between male WT and *Cc2d1a* cKO mice (Fig. 5B–D). In contrast, male *Cc2d1a* cKO mice showed a significant decrease in both mean mEPSC frequency and amplitude compared with WT mice (Fig. 5E–G). These results indicate that the loss of *Cc2d1a* in forebrain excitatory neurons decreases the excitability of PVN OXT neurons by reducing the synaptic excitation/inhibition (E/I) ratio.

**Fig. 6:**
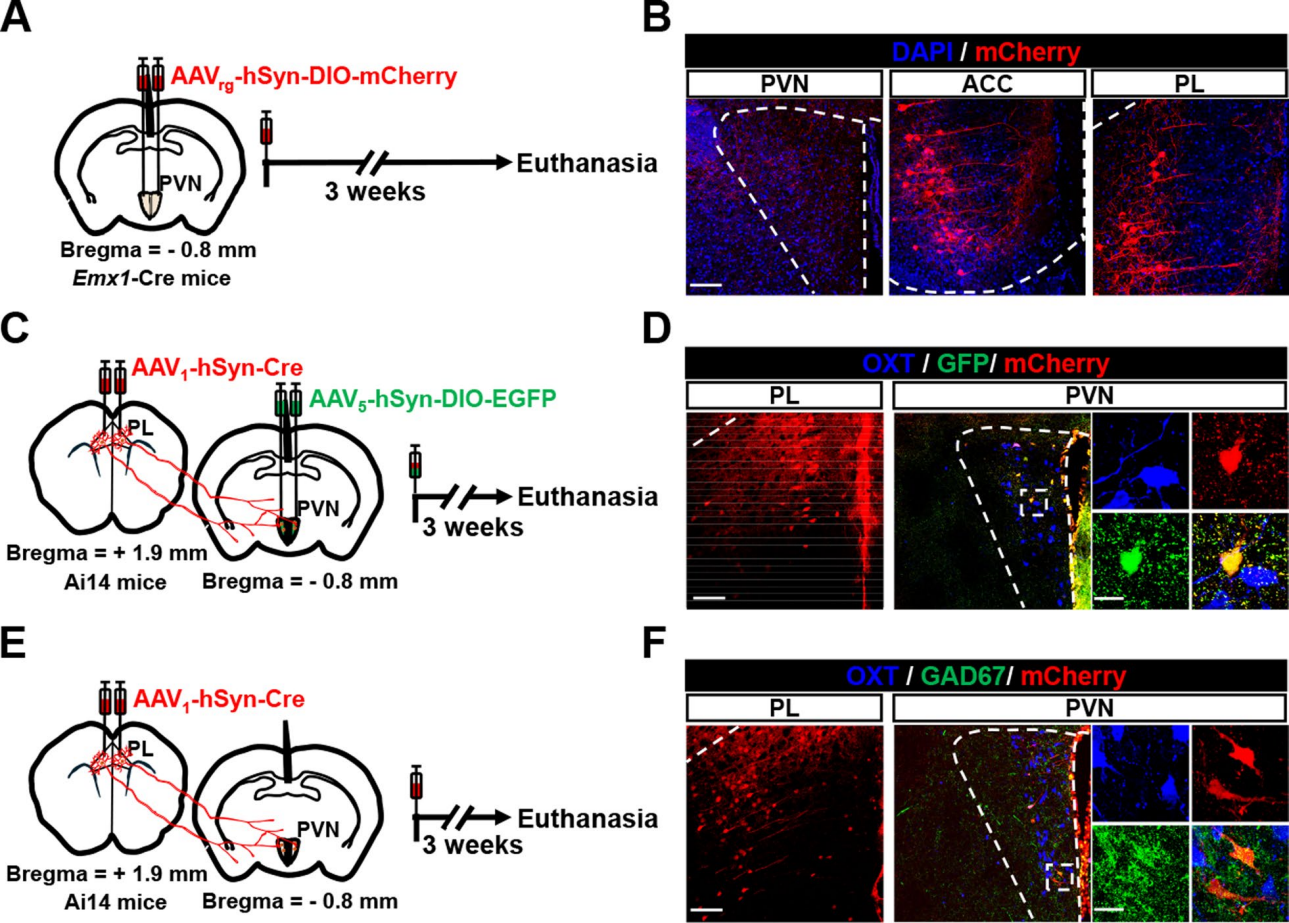
PrL glutamatergic neurons send an indirect projection to PVN OXT-expressing neurons via GABAergic interneurons. **A** Schematic diagram of the experimental design. AAVrg-hSyn-DIO-mCherry was injected bilaterally into the PVN of male *Emx1*-Cre mice. Three weeks later, the mice were euthanized, and their brain tissues were collected for immunohistochemical analysis. **B** Representative images showing the virus injection site in the PVN and retrogradely labeled neurons in the ACC and PrL. Scale bar, 100 μm. **C** Schematic diagram of the experimental design. AAV_1_-hSyn-Cre was injected bilaterally into the PrL, and AAV_5_-hSyn-DIO-EGFP was injected into the PVN of male Ai14 mice. Three weeks later, the mice were euthanized, and their brain tissues were collected for immunohistochemical analysis. **D** Representative images showing the virus injection site in the PrL and the expression of OXT-expressing and trans-synaptic neurons in the PVN. Scale bar, 100 μm. Magnified images of the rectangle show the OXT-expressing and trans-synaptic neurons. Scale bar, 20 µm. **E** Schematic diagram of the experimental design. AAV_1_-hSyn-Cre was injected bilaterally into the PrL of male Ai14 mice. Three weeks later, the mice were euthanized, and their brain tissues were collected for immunohistochemical analysis. **F** Representative images showing the virus injection site in the PrL and the expression of OXT-expressing neurons, GABAergic neurons, and transsynaptic neurons in the PVN. Scale bar, 100 μm. Magnified images of the rectangle reveal that GABAergic neurons, but not OXT-expressing neurons, co-express with transsynaptic neurons. Scale bar, 20 µm. Each experiment was replicated in 4 mice

Since *Emx1*-Cre mainly causes genetic recombination in forebrain excitatory neurons [31], we identified the forebrain region that might regulate PVN OXT neuron activity in response to *Cc2d1a* genetic ablation. To investigate potential upstream regions of PVN OXT neurons, male *Emx1*-Cre mice were injected with AAV_rg_-hSyn-DIO-mCherry into the PVN. Three weeks later, the animals were euthanized and examined for the distribution of forebrain cortical neurons that project to the PVN (Fig. 6A). We observed that neurons in the anterior cingulate cortex (ACC) and PrL send projections to the PVN (Fig. 6B). Given that male *Cc2d1a* cKO mice exhibit a significantly higher synaptic E/I ratio in the PrL [20], we focused on investigating the potential role of PrL projections in regulating PVN OXT neuron activity. For anterograde transsynaptic labeling of the specific downstream PVN neuron subtype that receives PrL projections, Ai14 mice were bilaterally injected with AAV_1_-hSyn-Cre into the PrL and with AAV_5_-hSyn-DIO-EGFP into the PVN (Fig. 6C). Three weeks later, we performed immunohistochemical analysis and observed that trans-synaptically labeled neurons (GFP^+^mCherry^+^) in the PVN were not OXT-immunoreactive (Fig. 6D). Using anterograde transsynaptic labeling and double immunohistochemical staining, we confirmed that the mCherry-labeled neurons in the PVN co-localize with neurons labeled by GAD67, a marker for inhibitory GABAergic neurons (Fig. 6E, F).

**Fig. 7:**
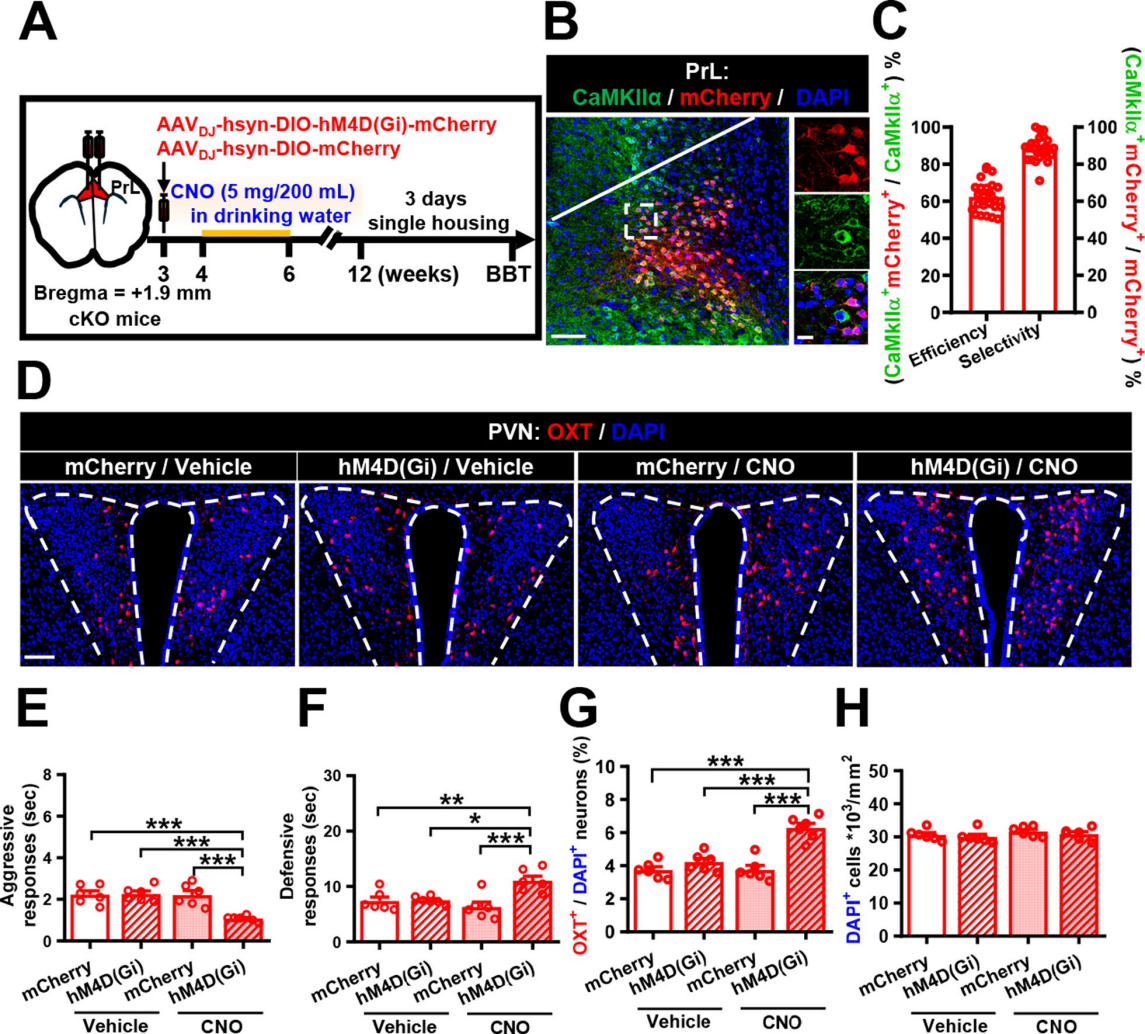
Chemogenetic inhibition of the PrL-PVN pathway alleviates irritability-like behavior in male *Cc2d1a* cKO mice. **A** Schematic diagram of the experimental timeline and design. AAV_DJ_-hSyn-DIO-hM4D(Gi)-mCherry or AAV_DJ_-hSyn-DIO-mCherry was injected bilaterally into the PrL of male *Cc2d1a* cKO mice at 3 weeks of age. One week after the viral infection, mice received drinking water containing either vehicle or CNO for 2 weeks. Their aggressive and defensive responses to the BBT were assessed at 12 weeks of age, after 3 days of individual housing. **B** Representative images showing the expression of CaMKIIα- and viral-expressing mCherry neurons in the PrL. Scale bar, 100 μm. **C** Percentage of CaMKIIα-expressing PrL neurons that also express hM4D(Gi)-mCherry (efficiency) as well as the percentage of mCherry-expressing PrL neurons that also express CaMKIIα (selectivity) (*N* = 24). **D** Representative images showing OXT-expressing neurons in the PVN. Scale bar, 100 μm. **E** Comparison of the time spent by male *Cc2d1a* cKO mice treated with vehicle or CNO in aggressive responses to the BBT (mouse numbers: *N*_mCherry/vehicle_ = 6, *N*_hM4D(Gi)/vehicle_ = 6, *N*_mCherry/CNO_ = 6, *N*_hM4D(Gi)/CNO_ = 6; two-way ANOVA, group: *F*_(1,20)_ = 11.84, *P* = 0.0026; treatment: *F*_(1,20)_ = 14.17, *P* = 0.0012; group × treatment interaction: *F*_(1,20)_ = 13.02, *P* = 0.0018). **F** Comparison of the time spent by *Cc2d1a* cKO mice treated with vehicle or CNO in defensive responses to the BBT (mouse numbers: *N*_mCherry/vehicle_ = 6, *N*_hM4D(Gi)/vehicle_ = 6, *N*_mCherry/CNO_ = 6, *N*_hM4D(Gi)/CNO_ = 6; two-way ANOVA, group: *F*_(1,20)_ = 11.34, *P* = 0.0031; treatment: *F*_(1,20)_ = 2.828, *P* = 0.1082; group × treatment interaction: *F*_(1,20)_ = 10.03, *P* = 0.0048). **G** Quantification of the percentage of OXT^+^/DAPI^+^ neurons (mouse numbers: *N*_mCherry/vehicle_ = 6, *N*_hM4D(Gi)/vehicle_ = 6, *N*_mCherry/CNO_ = 6, *N*_hM4D(Gi)/CNO_ = 6; two-way ANOVA, group:* F*_(1,20)_ = 36.82, *P* < 0.0001; treatment: *F*_(1,20)_ = 17.52, *P* = 0.0005; group × treatment interaction: *F*_(1,20)_ = 16.97, *P* = 0.0005) in male *Cc2d1a* cKO mice treated with vehicle or CNO. **H** Quantification of total DAPI^+^ cells (mouse numbers: *N*_mCherry/vehicle_ = 6, *N*_hM4D(Gi)/vehicle_ = 6, *N*_mCherry/CNO_ = 6, *N*_hM4D(Gi)/CNO_ = 6; two-way ANOVA, group: *F*_(1,20)_ = 0.9233, *P* = 0.2004; treatment: *F*_(1,20)_ = 1.753, *P* = 0.2004; group × treatment interaction: *F*_(1,20)_ = 0.018, *P* = 0.8946) in male *Cc2d1a* cKO mice treated with vehicle or CNO. Data are presented as mean ± SEM. *P < 0.05, **P < 0.01 and ***P < 0.001

We then asked whether chemogenetic suppression of the PrL-PVN pathway could improve irritability-like behavior in male *Cc2d1a* cKO mice. To examine this possibility, we bilaterally injected AAV_DJ_-hSyn-DIO-hM4D(Gi)-mCherry or AAV_DJ_-hSyn-DIO-mCherry into the PrL of male *Cc2d1a* cKO mice at 3 weeks of age. One week after the viral infection, mice were given drinking water containing either vehicle or CNO (5 mg/200 mL) for 2 weeks, and their aggressive and defensive responses to the BBT were assessed at 12 weeks of age, after being individually housed for 3 days (Fig. 7A). We verified the neuronal specificity of viral expression by imaging mCherry, whose expression was confined to CaMKIIα-immunoreactive neurons in the PrL (Fig. 7B). High transduction efficiency and selectivity were indicated by colocalization of mCherry and CaMKIIα (Fig. 7C). We also confirmed the OXT-expressing neurons in the PVN (Fig. 7D). A two-way ANOVA analysis on aggressive responses revealed a significant effect of DREADD, CNO and interaction (Fig. 7E). Post hoc analysis showed that chronic CNO treatment reduced aggressive responses in hM4D(Gi)-mCherry-expressed *Cc2d1a* cKO mice. A two-way ANOVA analysis on defensive responses also revealed significant effects of DREADD, CNO, and interaction (Fig. 7F). Post hoc analysis showed that CNO treatment reduced defensive response in hM4D(Gi)-mCherry-expressed *Cc2d1a* cKO mice. Consistently, we observed a significant increase in the percentages of OXT^+^/DAPI^+^ in the PVN of CNO-treated hM4D(Gi)-mCherry-expressing *Cc2d1a* cKO mice compared with vehicle-treated hM4D(Gi)-mCherry-expressing *Cc2d1a* cKO mice (Fig. 7G), whereas the total cell numbers of DAPI^+^ cells showed no difference between groups (Fig. 7H).

**Fig. 8:**
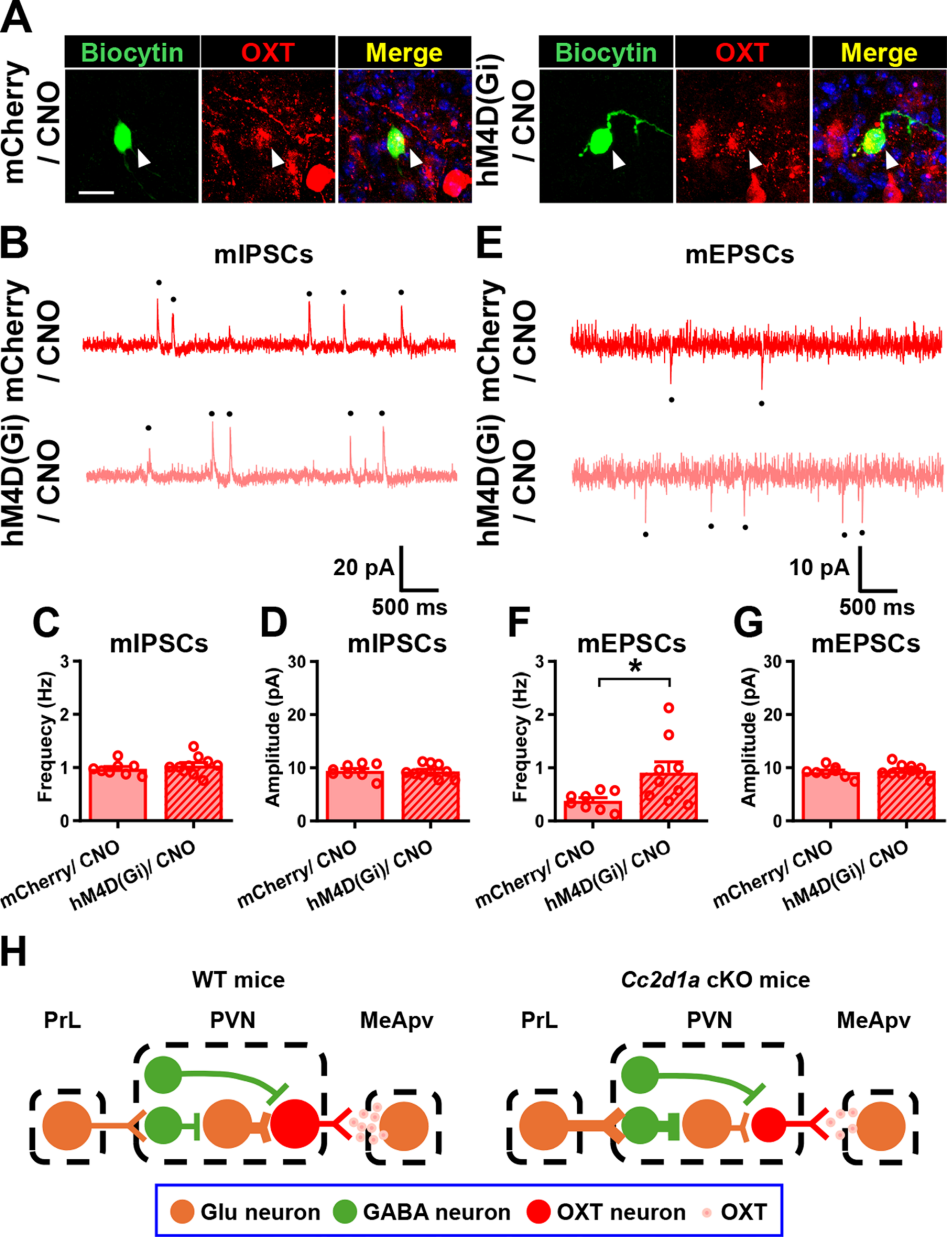
Chronic inhibition of the PrL-PVN pathway in *Cc2d1a* cKO mice restores normal synaptic transmission onto PVN OXT-expressing neurons. **A** Representative image of biocytin-labeled recorded OXT neurons in the PVN. Scale bar, 20 μm. **B** Representative traces of mIPSCs in PVN OXT neurons from mCherry/CNO- and hM4D(Gi)/CNO-treated groups of male C*c2d1a* cKO mice. **C**, **D** Comparison of the mean frequency (Mann–Whitney *U*-test, *P* = 0.3599; mCherry/CNO, *n* = 8 neurons from 3 mice, hM4D(Gi)/CNO, *n* = 10 neurons from 3 mice, **C**) and amplitude (Mann–Whitney *U*-test, *P* > 0.9999, **D**) of mIPSCs in PVN OXT neurons from mCherry/CNO- and hM4D(Gi)/CNO-treated groups of male *Cc2d1a* cKO mice. **E** Representative traces of mEPSCs in PVN OXT neurons from mCherry/CNO- and hM4D(Gi)/CNO-treated groups of male C*c2d1a* cKO mice. **F**, **G** Comparison of the mean frequency (Mann–Whitney *U*-test, *P* = 0.0206; mCherry/CNO, *n* = 8 neurons from 3 mice, hM4D(Gi)/CNO, *n* = 9 neurons from 3 mice, **F**) and amplitude (Mann–Whitney *U*-test, *P* = 0.7618, **G**) of mEPSCs in PVN OXT neurons from mCherry/CNO- and hM4D(Gi)/CNO-treated groups of male *Cc2d1a* cKO mice. **H** Proposed neural network mechanisms underlying the loss of Cc2d1a in forebrain excitatory neurons decrease the excitability of PVN OXT neurons. Loss of *Cc2d1a* in PrL-PVN projection neurons increases local GABAergic inhibition, thereby suppressing presynaptic glutamatergic inputs to PVN OXT neurons within glutamatergic microcircuits and reducing their activity. A subpopulation of GABAergic interneurons that synapse onto PVN neurons either does not receive PrL inputs or functions independently of them. Data are presented as mean ± SEM. *P < 0.05 as compared with the mCherry/CNO-treated group

To evaluate whether chemogenetic suppression of the PrL-PVN pathway restores the decreased synaptic E/I ratio onto PVN OXT neurons in male *Cc2d1a* cKO mice, we examined mIPSCs and mEPSCs. Biocytin labeling was used to confirm the identity of the recorded neurons (Fig. 8A). We observed no significant differences in the mean mIPSC frequency and amplitude between CNO-treated mCherry- and hM4D(Gi)-mCherry-expressing PVN OXT neurons of *Cc2d1a* cKO mice (Fig. 8B–D). Compared to CNO-treated mCherry-expressing PVN OXT neurons in *Cc2d1a* cKO mice, we observed that CNO-treated hM4D(Gi)-mCherry-expressing PVN OXT neurons in *Cc2d1a* cKO mice showed a significant increase in the average frequency of mEPSCs, with no change in the amplitude of mEPSCs (Fig. 8E–G). Altogether, these results suggest that PrL may indirectly regulate PVN OXT neuron activity by modulating local GABAergic and glutamatergic microcircuits rather than by direct, exclusive excitatory control (Fig. 8H).

### Ovariectomized female *Cc2d1a* cKO mice show increased irritability-like behavior associated with decreased AVP in the PVN

**Fig. 9:**
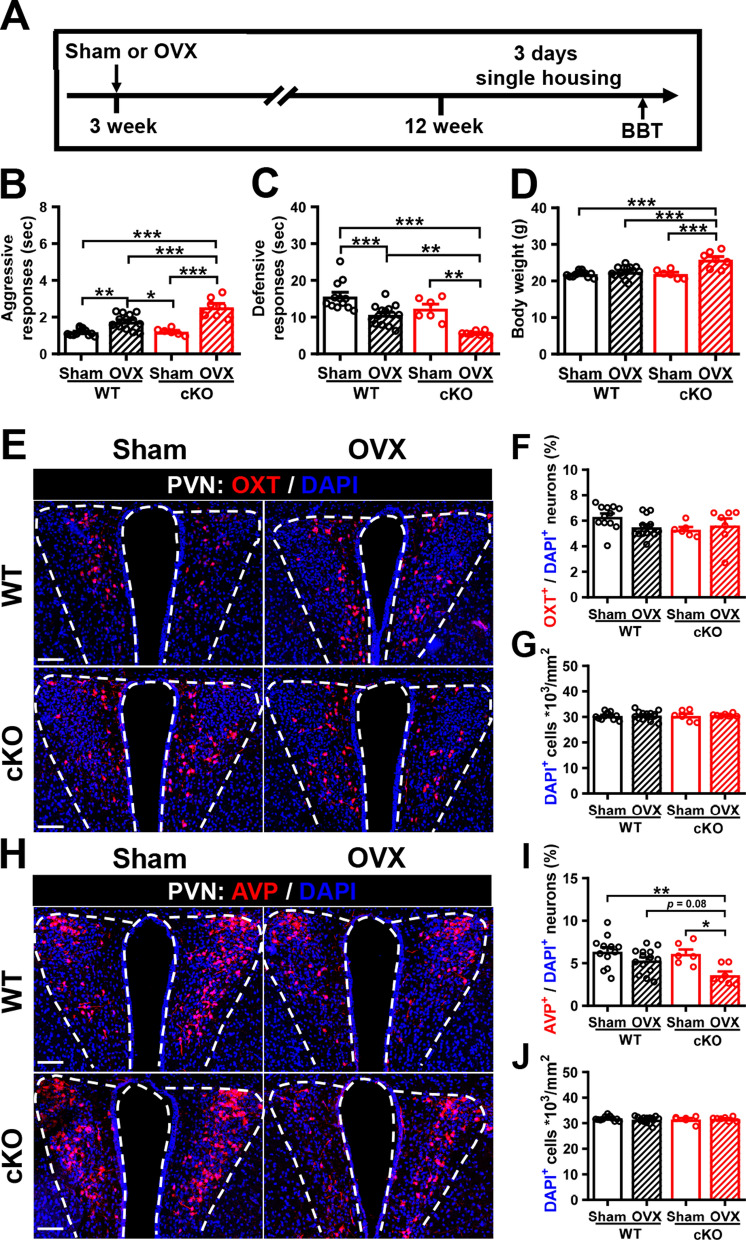
Ovariectomized female *Cc2d1a* cKO mice exhibited increased irritability-like behaviors and reduced vasopressin-expressing neurons in the PVN. **A** Schematic diagram of the experimental design. Female WT or *Cc2d1a* cKO mice underwent ovariectomy or a sham procedure at 3 weeks old, then were subjected to the BBT followed by three days of single housing. **B** Comparison of aggressive responses in the BBT (mouse numbers: *N*_WT-Sham_ = 12, *N*_WT-OVX_ = 14, *N*_cKO-Sham_ = 6, *N*_cKO-OVX_ = 7; two-way ANOVA, group: *F*_(1,35)_ = 11.01, *P* = 0.0021; treatment: *F*_(1,35)_ = 59.9, *P* < 0.0001; group × treatment interaction: *F*_(1,35)_ = 9.57, *P* = 0.0039). **C** Comparison of defensive responses in the BBT (mouse numbers: *N*_WT-Sham_ = 12, *N*_WT-OVX_ = 14, *N*_cKO-Sham_ = 6, *N*_cKO-OVX_ = 7; two-way ANOVA, group: *F*_(1,35)_ = 16.64, *P* = 0.0002; treatment: *F*_(1,35)_ = 32.76, *P* < 0.0001; group × treatment interaction: *F*_(1,35)_ = 0.67, *P* = 0.4187). **D** Comparison of body weight among each groups (mouse numbers: *N*_WT-Sham_ = 12, *N*_WT-OVX_ = 14, *N*_cKO-Sham_ = 6, *N*_cKO-OVX_ = 7; two-way ANOVA, group: *F*_(1,35)_ = 11.10, *P* = 0.0020; treatment: *F*_(1,35)_ = 20.61, *P* < 0.0001; group × treatment interaction: *F*_(1,35)_ = 10.55, *P* = 0.0026). **E** Representative images showing OXT-expressing neurons in the PVN of sham or OVX female WT or *Cc2d1a* cKO mice. Scale bar: 100 μm. **F**, **G** Bar graph showing the percentage of OXT^+^/DAPI^+^ neurons (mouse numbers: *N*_WT-Sham_ = 12, *N*_WT-OVX_ = 14, *N*_cKO-Sham_ = 6, *N*_cKO-OVX_ = 7; two-way ANOVA, group: *F*_(1,35)_ = 1.76, *P* = 0.1933; treatment: *F*_(1,35)_ = 0.5372, *P* = 0.4685; group × treatment interaction: *F*_(1,35)_ = 3.111, *P* = 0.0865; **F**) and the total number of DAPI^+^ cells (mouse numbers: *N*_WT-Sham_ = 12, *N*_WT-OVX_ = 14, *N*_cKO-Sham_ = 6, *N*_cKO-OVX_ = 7; two-way ANOVA, group: *F*_(1,35)_ = 0.0176, *P* = 0.8953; treatment: *F*_(1,35)_ = 0.2439, *P* = 0.6245; group × treatment interaction: *F*_(1,35)_ = 0.0382, *P* = 0.8463; **G**) in the PVN of sham or OVX female WT or *Cc2d1a* cKO mice. **H** Representative images showing the AVP-expressing neurons in the PVN of sham or OVX female WT or *Cc2d1a* cKO mice. Scale bar: 100 μm. **I**, **J** Bar graph showing the percentage of AVP^+^/DAPI^+^ neurons (mouse numbers: *N*_WT-Sham_ = 12, *N*_WT-OVX_ = 14, *N*_cKO-Sham_ = 6, *N*_cKO-OVX_ = 7; two-way ANOVA, group: *F*_(1,35)_ = 3.66, *P* = 0.0639; treatment: *F*_(1,35)_ = 11.76, *P* = 0.0016; group × treatment interaction: *F*_(1,35)_ = 1.983, *P* = 0.1679; **I**) and the total number of DAPI^+^ cells (mouse numbers: *N*_WT-Sham_ = 12, *N*_WT-OVX_ = 14, *N*_cKO-Sham_ = 6, *N*_cKO-OVX_ = 7; two-way ANOVA, group: *F*_(1,35)_ = 0.0146, *P* = 0.9047; treatment: *F*_(1,35)_ = 0.1352, *P* = 0.7153; group × treatment interaction: *F*_(1,35)_ = 0.8574, *P* = 0.3608;** J**) in the PVN of sham or OVX female WT or *Cc2d1a* cKO mice. Data are presented as mean ± SEM. *P < 0.05, **P < 0.01, and ***P < 0.001

Our previous study found that irritability-like behavior is less pronounced in female *Cc2d1a* cKO mice [18]. Because the ovarian hormone estrogen has been strongly implicated in regulating mood and behavior [32], we tested whether it protects against irritability-like behavior in female *Cc2d1a* cKO mice. To understand the role of estrogen in sex differences in the ability to display irritability-like behavior in *Cc2d1a* cKO mice, we examined aggressive and defensive responses to the BBT in ovariectomized (OVX) female *Cc2d1a* cKO mice. Sham or OVX procedures were performed on female WT or *Cc2d1a* cKO mice at 3 weeks of age, and their aggressive and defensive responses to the BBT were assessed at 12 weeks of age, after being individually housed for 3 days (Fig. 9A). A two-way ANOVA on aggressive responses revealed significant effects of genotype, OVX, and their interaction (Fig. 9B). Post hoc analysis showed that OVX treatment increased aggressive responses in female *Cc2d1a* cKO mice. Additionally, a two-way ANOVA of defensive responses revealed significant effects of genotype, OVX, and their interaction (Fig. 9C). Post hoc analysis showed that OVX treatment reduced defensive responses in female *Cc2d1a* cKO mice. Notably, compared to the Sham group, OVX female *Cc2d1a* cKO mice exhibited a significant increase in body weight (Fig. 9D). Surprisingly, we found no significant differences in the percentage of OXT-immunoreactive neurons in the PVN between OVX and Sham female *Cc2d1a* cKO mice (Fig. 9E, F). The total number of DAPI^+^ cells in the PVN did not differ across groups (Fig. 9G). By contrast, we found a significant decrease in the percentage of AVP-immunoreactive neurons in the PVN of OVX female *Cc2d1a* cKO mice compared with sham female *Cc2d1a* cKO mice (Fig. 9H, I), whereas no significant difference was observed among groups in the total number of DAPI^+^ cells in the PVN (Fig. 9J). These results suggest that decreased AVP, but not OXT, expression in the PVN is associated with increased irritability-like behavior in OVX female *Cc2d1a* cKO mice.

## Discussion

Although increased irritability commonly co-occurs with ASD, few studies have examined the neural mechanisms underlying irritability in ASD. Our previous study showed dysregulation of the PVN OXT system in male *Cc2d1a* cKO mice and provided evidence that endogenous and evoked OXT can reduce irritability-like behavior [18]. In this study, we examined how deleting *Cc2d1a* in forebrain excitatory neurons reduces OXT expression in the PVN, thereby increasing irritability-like behavior. We demonstrated that adult male *Cc2d1a* cKO mice exhibit reduced OXT expression in both magnocellular and parvocellular PVN neurons. Specifically, we identified a neural pathway from the PrL to the PVN that plays a crucial role in indirectly regulating OXT neuron activity by modulating local GABAergic and glutamatergic microcircuits. Our results emphasize that lower OXT expression is associated with reduced activity of PVN OXT neurons due to an alteration in the synaptic E/I balance within these neurons. Surprisingly, OVX female *Cc2d1a* cKO mice exhibit increased irritability-like behavior, associated with reduced AVP expression in the PVN. Collectively, our findings provide mechanistic insights into how the dysregulated PVN OXT system contributes to ASD-related irritability.

Central OXT system disruptions have been consistently observed across multiple ASD mouse models, manifesting as deficits in social behavior, altered social recognition, and impaired communication [11–17]. Our previous study clearly demonstrated an inverse relationship between reduced PVN OXT expression and increased irritability-like behavior in male *Cc2d1a* cKO mice [18]. To clarify the developmental stage at which the reduction in OXT-expressing neurons begins in male *Cc2d1a* cKO mice, we found that the decline is evident at 12 and 24 weeks of age. This finding aligns with previous studies showing that the development of OXT-expressing neurons continues after birth and can be modified throughout life [17, 33, 34]. Surprisingly, qPCR analysis showed no change in *Oxt* mRNA transcription in the PVN of *Cc2d1a* cKO mice compared with WT mice. Our current study did not examine why *Oxt* mRNA levels remain unchanged while protein expression decreases in the PVN of *Cc2d1a* cKO mice. The discrepancy between OXT protein and mRNA levels can be explained by several post-transcriptional and post-translational mechanisms that regulate protein synthesis, degradation, and secretion independently of mRNA expression. Future studies will be required to ascertain whether deleting *Cc2d1a* from forebrain excitatory neurons reduces PVN OXT expression through post-transcriptional regulation [35], translation-initiated mRNA decay [36], increased protein degradation [37], epigenetic changes [38], or altered peptide dynamics. However, our data suggest that the reduction in OXT-expressing neurons is not caused by a general loss of PVN neurons, as the total PVN cell count is comparable between male WT and *Cc2d1a* cKO mice.

How is the number of OXT-expressing neurons precisely controlled in the adult brain? One would expect neuronal activity to be a crucial factor. To investigate whether neuronal activity can directly influence OXT expression, we examined the effect of reduced excitability by selectively and stably expressing the inhibitory DREADDs in PVN OXT neurons and observed a significant decrease in the number of OXT-expressing neurons in adulthood after chronic chemogenetic silencing of PVN OXT neurons during adolescence. This suggests that a shift toward a more inhibitory state may be explained by decreased OXT expression in the PVN of male *Cc2d1a* cKO mice. This finding aligns with a previous study showing that relaxin-3 binds to RXFP3 receptors and activates an inhibitory outward current in PVN neurons, thereby reducing their activity and, consequently, OXT expression [39]. Our in vivo fiber photometry data also showed that male *Cc2d1a* cKO mice exhibit decreased OXT dynamics in the MeApv during the BBT, which strongly correlates with their aggressive and defensive responses. Our findings supporting an anti-irritability function of OXT align with a recent clinical study reporting that youth with irritability who received intranasal OXT showed significant reductions in irritability and reactive aggression [40].

A crucial balance between excitatory glutamate and inhibitory GABA synaptic transmission maintains neuronal excitability. The mechanisms governing the balance between glutamate and GABA signaling in the PVN remain poorly understood. Given that our previous study showed that *Emx1*-Cre-mediated recombination is restricted to forebrain excitatory neurons and does not directly affect *Cc2d1a* expression within PVN OXT neurons [18], we hypothesized that long-range glutamatergic inputs from forebrain structures may regulate the dynamic balance of synaptic excitation and inhibition in PVN OXT neurons, thereby governing OXT expression. Because male *Cc2d1a* cKO mice show a significantly higher synaptic E/I ratio in the PrL [20], we focused on the role of PrL projections in controlling PVN OXT neuron activity. Anatomical studies have not shown direct projections from the PrL to the PVN [41, 42]; however, using both retrograde and anterograde trans-synaptic viral tracing, we demonstrate that the PrL may indirectly regulate PVN OXT neuron activity by modulating local GABAergic and glutamatergic microcircuits rather than by direct excitatory control. Our findings indicate that *Cc2d1a* cKO mice exhibited a preferential decrease in the frequency and amplitude of mEPSCs in PVN OXT neurons, suggesting that an altered synaptic E/I balance, favoring decreased excitatory transmission, likely underlies the decreased activity of PVN OXT neurons observed in male *Cc2d1a* cKO mice and reduces OXT expression. Importantly, using projection-specific chemogenetic inhibition, we demonstrate that suppressing the PrL-PVN pathway restores the reduced synaptic E/I ratio onto PVN OXT neurons and increases OXT expression in male *Cc2d1a* cKO mice. Collectively, our results support a model in which loss of *Cc2d1a* in PrL-PVN projection neurons increases local GABAergic inhibition, thereby suppressing presynaptic glutamatergic inputs to OXT neurons within glutamatergic microcircuits, reducing neuronal activity and OXT expression. Recently, Yamaguchi et al. [43] showed that excess glutamate release can dynamically strengthen GABAergic synaptic inhibition of PVN neurons. Since our results show no increase in mIPSCs onto PVN OXT neurons in *Cc2d1a* cKO mice, it is unlikely that the reduced OXT expression results from increased GABAergic inhibition directly onto PVN OXT neurons. The lack of an effect on mIPSCs onto PVN OXT neurons in *Cc2d1a* cKO mice also reflects the possibility that a subpopulation of GABAergic interneurons that synapse onto PVN neurons either does not receive PrL inputs or operates independently of them. Furthermore, although our study focused specifically on the PrL, further research is needed to definitively rule out contributions from other forebrain excitatory neurons affected by *Cc2d1a* deletion.

*Cc2d1a* cKO mice display several key features of ASD, including impairments in social communication and interaction, as well as restricted and repetitive behaviors, with sex-specific differences [19, 44]. A similar male-biased behavioral deficit in *Cc2d1a* cKO mice was also observed in the BBT to assess irritability-like behavior [18]. Although the precise neural mechanisms underlying these sex-specific differences in susceptibility to *Cc2d1a* deletion remain to be identified, they may reflect compensatory mechanisms or inherent differences in the distribution of molecular and cellular functions between males and females [45]. Our current work complements these findings by showing that female *Cc2d1a* cKO mice display irritability-like behavior after OVX. Surprisingly, we found a decrease in AVP but not in OXT expression in the PVN of OVX female *Cc2d1a* cKO mice. These findings suggest that sex differences in irritability-like behavior may be evolutionarily preserved, with the OXT and AVP systems playing sex-specific roles in regulating it. Although we cannot rule out that dysregulation of the AVP system in males or the OXT system in females also causes irritability-like behavior, additional studies are needed to clarify the neural mechanisms underlying sex-specific differences in the OXT and AVP systems that regulate the emotional state of *Cc2d1a* cKO mice. Our data also indicate that chemogenetic suppression of the PrL-PVN pathway during adolescence restores PVN OXT expression and reduces irritability-like behavior in adult male *Cc2d1a* cKO mice. This suggests that early interventions may offer promising avenues to mitigate developmental consequences in *Cc2d1a* cKO mice, potentially providing therapeutic strategies to reduce the severity of associated ASD-like behaviors across the lifespan.

We recognize several limitations in our study. First, only the BBT was used to measure irritability-like behavior. Although the BBT is the only approved behavioral test for assessing irritability-like behavior [18, 46], it might not fully capture the complexity of irritability in ASD and may be subject to experimenter bias and scoring variability. We have used stringent, standardized protocols, such as blinding and independent raters, to ensure objective and consistent results, but other novel behavioral paradigms that elicit frustration, such as the alternate poking reward omission task, may be useful for measuring irritability [47]. Second, our data indicate that decreased AVP expression in the PVN is linked to increased irritability-like behavior in OVX female *Cc2d1a* cKO mice; however, it remains unclear whether this reduction also reflects a change in the synaptic E/I balance of PVN AVP neurons. Third, because social approaches and interactions can increase OXT expression [9, 48], we cannot exclude the possibility that dysregulation of the OXT system is partly linked to decreased social interactions in male *Cc2d1a* cKO mice [20]. Finally, it remains unclear whether similar mechanisms are at work across different genetic mouse models of ASD that exhibit loss of OXT neurons in the PVN [17, 49]. Further studies are warranted to resolve these issues.

## Conclusions

This study provides mechanistic insights into the reduction of OXT expression in the PVN of *Cc2d1a* cKO mice, thereby contributing to increased irritability-like behavior. We further identify a neural pathway from the PrL to the PVN that indirectly influences OXT neuron activity via GABAergic interneurons, establishing a causal connection between OXT deficiency and irritability-like behavior. In addition to showing increased irritability-like behavior [18], *Cc2d1a* cKO mice recapitulated numerous ASD-like behavioral phenotypes, including social deficits and increased repetitive behaviors [19, 20]. Our ongoing experiments also reveal that intranasal OXT administration effectively rescues social interaction deficits in the reciprocal social interaction test and impaired sociability and preference for social novelty in the three-chamber social interaction test, but not restricted repetitive behaviors such as excessive self-grooming and digging. Altogether, these findings provide insight into how ASD-risk genes interact with the PVN OXT system and suggest that OXT may have therapeutic value for treating comorbid social deficits and irritability in individuals with ASD due to *Cc2d1a* haploinsufficiency.

## Data Availability

The authors confirm that all data generated and analyzed during this study are either included in this published article or available from the corresponding authors upon reasonable request. All data produced or examined during this study are included in the published article.

## Abbreviations

AAV: Adeno-associated virus
ACC: Anterior cingulate cortex
aCSF: Artificial cerebrospinal fluid
ANOVA: Analysis of variance
AP: Anteroposterior
APV: 2-Amino-5-phosphonovaleric acid
ASD: Autism spectrum disorder
AUC: Area under the curve
AVP: Arginine vasopressin
BBT: Bottle-brush test
Cc2d1a: Coiled-coil and C2 domain containing 1a
cKO: Conditional knockout
CNO: Clozapine-*N*-oxide
CNQX: 6-Cyano-7-nitroquinoxaline-2,3-dione
DAPI: 4',6-Diamidino-2-phenylindole
DMSO: Dimethyl sulfoxide
DREADD: Designer receptors exclusively activated by designer drugs
DV: Dorsoventral
E/I: Excitation/inhibition
FG: Fluoro-Gold
GABA: γ-Aminobutyric acid
GAD67: Glutamic acid decarboxylase 67
GAPDH: Glyceraldehyde-3-phosphate dehydrogenase
IACUC: Institutional Animal Care and Use Committee
LED: Light-emitting diode
MeApv: Posteroventral medial amygdala
mEPSC: Miniature excitatory postsynaptic current
mIPSC: Miniature inhibitory postsynaptic current
ML: Mediolateral
OXT: Oxytocin
OVX: Ovariectomy
PCR: Polymerase chain reaction
PFA: Paraformaldehyde
PrL: Prelimbic cortex
PVN: Paraventricular nucleus of the hypothalamus
qPCR: Quantitative polymerase chain reaction
SEM: Standard error of the mean
SON: Supraoptic nucleus
TTX: Tetrodotoxin
WT: Wild-type
